# Effects of cardiac telerehabilitation in patients with coronary artery disease using a personalised patient-centred web application: protocol for the SmartCare-CAD randomised controlled trial

**DOI:** 10.1186/s12872-017-0477-6

**Published:** 2017-01-31

**Authors:** Rutger W. M. Brouwers, Jos J. Kraal, Simone C. J. Traa, Ruud F. Spee, Laurence M. L. C. Oostveen, Hareld M. C. Kemps

**Affiliations:** 1 Department of Cardiology, Máxima Medical Centre, Eindhoven/Veldhoven, The Netherlands; 2 FLOW Centre for Rehabilitation and Prevention in chronic disease, Máxima Medical Centre, Eindhoven/Veldhoven, The Netherlands; 3 Department of Medical Psychology, Máxima Medical Centre, Eindhoven/Veldhoven, The Netherlands

**Keywords:** Cardiac rehabilitation, Telerehabilitation, Behavioural change, Physical activity, Patient empowerment, Cost-effectiveness

## Abstract

**Background:**

Cardiac rehabilitation has beneficial effects on morbidity and mortality in patients with coronary artery disease, but is vastly underutilised and short-term improvements are often not sustained. Telerehabilitation has the potential to overcome these barriers, but its superiority has not been convincingly demonstrated yet. This may be due to insufficient focus on behavioural change and development of patients’ self-management skills. Moreover, potentially beneficial communication methods, such as internet and video consultation, are rarely used. We hypothesise that, when compared to centre-based cardiac rehabilitation, cardiac telerehabilitation using evidence-based behavioural change strategies, modern communication methods and on-demand coaching will result in improved self-management skills and sustainable behavioural change, which translates to higher physical activity levels in a cost-effective way.

**Methods:**

This randomised controlled trial compares cardiac telerehabilitation with centre-based cardiac rehabilitation in patients with coronary artery disease. We randomise 300 patients entering cardiac rehabilitation to centre-based cardiac rehabilitation (control group) or cardiac telerehabilitation (intervention group). The core component of the intervention is a patient-centred web application, which enables patients to adjust rehabilitation goals, inspect training and physical activity data, share data with other caregivers and to use video consultation. After six supervised training sessions, the intervention group continues exercise training at home, wearing an accelerometer and heart rate monitor. In addition, physical activity levels are assessed by the accelerometer for four days per week. Patients upload training and physical activity data weekly and receive feedback through video consultation once a week. After completion of the rehabilitation programme, on-demand coaching is performed when training adherence or physical activity levels decline with 50% or more. The primary outcome measure is physical activity level, assessed at baseline, three months and twelve months, and is calculated from accelerometer and heart rate data. Secondary outcome measures include physical fitness, quality of life, anxiety and depression, patient empowerment, patient satisfaction and cost-effectiveness.

**Discussion:**

This study is one of the first studies evaluating effects and costs of a cardiac telerehabilitation intervention comprising a combination of modern technology and evidence-based behavioural change strategies including relapse prevention. We hypothesise that this intervention has superior effects on exercise behaviour without exceeding the costs of a traditional centre-based intervention.

**Trial registration:**

Netherlands Trial Register NTR5156. Registered 22 April 2015.

**Electronic supplementary material:**

The online version of this article (doi:10.1186/s12872-017-0477-6) contains supplementary material, which is available to authorized users.

## Background

Cardiac rehabilitation (CR) has proven beneficial effects on morbidity and mortality, and is highly recommended in clinical guidelines for patients with coronary artery disease (CAD) [1–4]. Unfortunately, CR is vastly underutilised, due to low referral rates and patient-related factors such as travelling distance, work, or social obligations [5, 6]. In addition, improvements in lifestyle behaviour are often not maintained over time. As such, physical activity levels often decline after a successful rehabilitation programme and a significant amount of patients with CAD do not meet the targets for secondary prevention of cardiovascular disease (lifestyle, risk factor and therapeutic targets) [7–9].

Alternatives for current centre-based CR should not only aim to improve uptake rates, but also to maintain the beneficial effects in the long term. For this purpose, it may be effective to shift (part of) the rehabilitation programme to the home environment, especially for patients who are unable to participate in conventional centre-based CR due to transport difficulties or work resumption. A meta-analysis showed no difference between home-based and centre-based CR in mortality risk, cardiac events, improvement of exercise capacity and modifiable risk factors [10]. However, these home-based interventions often lack long-term follow-up and applied varying monitoring strategies and training protocols in heterogeneous study populations. Studies that do report long-term effects showed inconsistent results. A study by Smith et al. showed a decline in peakVO_2_ between one and six years follow-up in both the home-based and hospital-based group, but the rate of decline was significantly smaller in the home-based group [11]. Dracup et al. showed a decline in peakVO_2_ after an initial improvement in patients with heart failure following a home-based walking programme and resistance training [12]. A decline in functional performance might be explained by the fact that these home-based interventions did not primarily focus on behavioural change or on development of self-management skills but on exercise training. In fact, behavioural aspects such as dietary intake and activity behaviour were not monitored.

A more comprehensive approach is provided by cardiac telerehabilitation, which often incorporates multiple components of CR and uses monitoring devices and remote communication with patients to deliver CR outside the hospital environment. Providing objective feedback and allowing patients to track their own progress may increase patients’ self-management skills and thus establish a sustainable behavioural change [13–15]. Two recent systematic reviews reported that, compared to centre-based CR, telerehabilitation resulted in fewer adverse events and rehospitalisation, higher physical activity levels, better adherence to physical activity guidelines, and improved LDL-cholesterol and diastolic blood pressure levels [16, 17]. However, two other systematic reviews showed no differences in outcomes in mortality or modifiable risk factors [18, 19]. The majority of telerehabilitation interventions for cardiac patients are telephone-based interventions, while other methods of communication (e.g. internet, video consultation and/or text messages) may enhance the provision of objective feedback [20]. Furthermore, the effectiveness of internet-based behavioural change interventions increases when one or more multiple behaviour change techniques are applied [20, 21]. For instance, relapse prevention is a cognitive behavioural strategy that can be used in (internet-based) behavioural change interventions such as cardiac telerehabilitation. Relapse prevention teaches an individual how to identify and cope with situations in which the actual behaviour deviates from the intended behaviour. It can be applied to multiple treatment modules in telerehabilitation (e.g. nutritional counselling, smoking cessation or physical activity coaching). Other strategies include goal setting, self-monitoring, provision of feedback, enhancement of self-efficacy and motivational interviewing [22]. If these techniques are implemented, we expect that they will lead to a more sustainable improvement of cardiovascular risk profiles.

We hypothesize that cardiac telerehabilitation using modern communication methods and multiple evidence-based behavioural change strategies including relapse prevention, will result in improved self-management skills and sustainable behavioural change. We expect this will translate to a superior increase in physical activity levels when compared to centre-based CR. Therefore, the objective of the SmartCare-CAD trial is to investigate whether cardiac telerehabilitation using modern technology and multiple behavioural change strategies results in better long-term physical activity levels than centre-based CR in patients with CAD. For telerehabilitation, a personalised patient-centred web application is used, comprising remote monitoring of exercise and physical activity behaviour and on-demand video consultation. In addition, both strategies will be compared with respect to physical fitness, modifiable cardiovascular risk factors, quality of life, anxiety and depression disorders, patient empowerment, patient satisfaction and cost effectiveness.

## Methods

### Study design

This study is designed as a monocentre randomised controlled trial at Máxima Medical Centre Eindhoven/Veldhoven, the Netherlands. Patients from both Máxima Medical Centre and Catharina Hospital Eindhoven are included in the trial. Three hundred patients with CAD entering outpatient CR are randomly allocated to cardiac telerehabilitation with home-based exercise training (intervention group) or centre-based CR (control group). All subjects are requested to provide written informed consent before study entry. Data are collected at baseline and after three, six, nine, and twelve months. The protocol for this study was approved by the Institutional Review Board of Máxima Medical Centre Veldhoven in the Netherlands. The trial is registered at the Netherlands Trial Registry (NTR) with registration number NTR5156. A completed SPIRIT (Additional file 1: Standard Protocol Items: Recommendations for Interventional Trials) diagram can be found in Table 1.

**Table 1:**
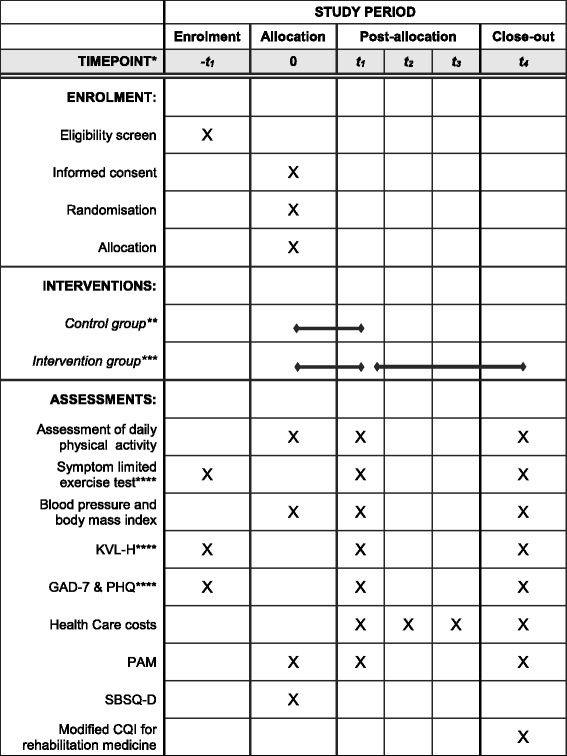
Completed SPIRIT diagram for SmartCare-CAD

* t_1_ = three months, t_2_ = six months, t_3_ = nine months, t_4_ = twelve months

** Three months

** Three months + nine months of on-demand coaching

**** Part of routine care and therefore assessed before informed consent

*KVL-H* Kwaliteit van Leven bij Hartpatiënten, *GAD-7* Generalized Anxiety Disorder, *PHQ* Patient Health Questionnaire, *PAM* Patient Activation Measure, *SBSQ-D* Set of Brief Screening Questions – Dutch, *CQI* Consumer Quality Index

### Study population

Patients entering outpatient CR because of CAD are considered for participation; i.e., patients with stable CAD, an acute coronary syndrome (with or without ST-segment elevation) and/or after coronary revascularization (primary or elective percutaneous coronary intervention (PCI) or coronary artery bypass grafting (CABG)). Only patients referred to exercise training as a part of outpatient CR, based on the individual needs assessment from the guidelines on outpatient CR of the Dutch Society of Cardiology, are asked to participate [23]. Eligible patients are at least 18 years old and have access to a personal computer with Internet connectivity at home. Patients are required to have a mobile phone with short message service (SMS) functionality to login to the web application with two-factor authentication. Patients that do not have a personal computer can be provided with a tablet that has necessary software pre-installed. Exclusion criteria are: 1) ventricular arrhythmia or myocardial ischemia during low to moderate exercise intensity as assessed by symptom limited exercise testing at baseline; 2) heart failure NYHA (New York Heart Association) class IV, and 3) comorbidity precluding exercise training (e.g. orthopaedic, neurological or cognitive conditions). A complete list of inclusion and exclusion criteria is provided in Table 2.

**Table 2:** Inclusion and exclusion criteria for SmartCare-CAD

Inclusion criteria	
i.	Referral for cardiac rehabilitation due to stable angina pectoris, acute coronary syndrome (with or without ST-segment elevation) or after coronary revascularization, i.e. (primary or elective) PCI or CABG
ii.	Indication for exercise training as a part of outpatient cardiac rehabilitation
iii.	Personal computer with internet access at home
iv.	Possession of a mobile phone with SMS-functionality
v.	Age ≥18 years
vi.	Able to speak, read and write Dutch
Exclusion criteria	
i.	Ventricular arrhythmia or myocardial ischemia during low to moderate exercise intensity as assessed by symptom limited exercise testing at baseline
ii.	Heart failure NYHA class IV
iii.	Comorbidity precluding exercise training (e.g. orthopaedic, neurological or cognitive conditions)

*PCI* percutaneous coronary intervention, *CABG* coronary artery bypass grafting, *SMS* Short Message Service, *NYHA* New York Heart Association

### Randomisation, blinding and treatment allocation

Patients are randomly allocated to the intervention or control group after baseline measurements by the investigator using a computerised randomisation system in the web-based database software Castor EDC (Castor Electronic Data Capture, Ciwit BV, Amsterdam, The Netherlands), in which block randomisation with variable block size (4, 6 or 8) is applied. Randomisation is stratified for gender and left ventricular ejection fraction (LVEF ≤ 35% or LVEF > 35%) to ensure balance of the treatment arms with respect to the combinations of the prognostic variables. The investigator, supervising healthcare professionals and patients are not blinded for treatment due to the nature of the intervention.

### Sample size calculation

Adopted from Kraal et. al [24], we calculated sample size for the primary outcome measure –physical activity energy expenditure (PAEE) – assuming PAEE in healthy subjects was 4.0 ± 1.2 MJ/day [25]. If the difference in the primary outcome measure between the intervention and control groups is 10%, 143 subjects need to be included in either group (power = 0.8 and alpha =0.05). Taking into account loss to follow-up, 150 subjects are included in both groups.

### Exercise training programme

The CR programme may consist of the following supervised outpatient treatment modules: exercise training, an information programme, a relaxation programme, a smoking cessation programme, a psycho-educative prevention programme and/or individual treatment by a psychologist or dietician. Based on the individual needs assessment procedure, one or more of these treatment modules are selected [23]. Treatment goals and results of the needs assessment are registered in the Hospital Information System by the nurse specialist. In both treatment groups, the CR programme consists of at least the exercise training module. The exercise training programme in both the intervention and control group is composed according to evidence-based clinical algorithms for the prescription and evaluation of exercise-based CR [26]. These algorithms serve as best practice standards in the Netherlands and were developed using recent Dutch and European CR guidelines and position statements. The content of the programme is determined individually, based on the referral diagnose, training goal(s) and physical fitness level. The programme may consist of several training modalities, including aerobic training (continuous training (CT) or high-intensity interval training (HIT)), functional training and resistance training. In aerobic training, the training frequency ranges from 2–5 sessions per week, lasting 20–60 min at 50–80% (CT) or 80–90% (HIT) of the heart rate reserve (HRR). We expect aerobic training to be similar in both treatment groups in terms of duration, frequency and intensity.

### Control group (centre-based cardiac rehabilitation)

Patients in the control group participate in group-based training sessions at the outpatient clinic (group size varies between 8 to 12 participants) under supervision of physical therapists and exercise specialists. All physical therapists and exercise specialists involved in this trial are specialised in CR and have been trained in motivational interviewing. Patients receive an individually tailored training programme on a treadmill or an electromagnetically braked cycle ergometer. Therefore, the total number of training sessions will vary among patients according to the clinical algorithms [26]. The physical therapist documents training attendance. In the last training session, the training programme is evaluated and patients are encouraged to continue physical activities at home. After 3 months, all treatment modules of the CR programme are evaluated by a nurse specialist at the outpatient clinic.

### Intervention group (cardiac telerehabilitation)

#### Web application

The core component of the study intervention is a secured, personalised, patient-centred web application. Safety and privacy are warranted by using encryption and signature layers. This platform enables patients to:

- Register and adjust rehabilitation goals, training goals and treatment modules
- Upload and review exercise training data
- Upload and review daily physical activity data
- Perform video consulting with physical therapists
- Permit relevant pre-specified caregivers (e.g. cardiologist, psychologist, general practitioner or informal carers) to have access to the abovementioned data.

After the randomisation procedure, patients select and specify their treatment goals during the second part of the intake procedure with the nurse specialist. These goals will be registered in the web application by the patient and the investigator. During this meeting patients are instructed to use the platform on their personal computer, and to provide access to other caregivers. Patients also receive a heart rate monitor (Mio Alpha, Physical Enterprises Inc., Vancouver, British Columbia, Canada) and accelerometer (ActiGraph wGT3x-BT, Actigraph LLC, Pensacola, Florida, USA).

#### Exercise training

Patients in the intervention group start their CR programme with six supervised training sessions in the outpatient clinic, similar to patients in the control group. During these sessions, they are instructed about (home-based) training and training intensity, the use of their heart rate monitor and accelerometer and the transfer of data to the web application. In addition, patients are counselled on the training modality (e.g. cycling, walking/running) they prefer to incorporate in their exercise programme at home.

After six training sessions, exercise training is transferred to the home environment. Exercise training is continued in the hospital when home-based training is considered unsafe by the CR cardiologist or when the patient prefers to continue training in the hospital. If exercise training is transferred to the home environment, exercise training targets (i.e. frequency, session duration and time in heart rate zone) and physical activity targets (time spent at a moderate or high activity level, using a modified version of the algorithm proposed by Sasaki et al. [27]) are recorded in the web application. A weekly video consultation with the physical therapist is scheduled until CR is completed and evaluated at the outpatient clinic after three months. During these video consultations, both the exercise data and physical activity data are evaluated and targets are adjusted if needed.

If exercise training is not transferred to the home environment, patients continue exercise training at the outpatient clinic. Similar to the control group, the total number of training sessions is based on the clinical algorithms. However, physical activity is monitored at home, using data from the accelerometer. During the exercise training sessions at the hospital, exercise and physical activity targets are evaluated, and adjusted in the web application if needed.

#### Telemonitoring guidance

Patients are instructed to wear a wrist-worn heart rate monitor with a display (Mio Alpha) and a hip-worn accelerometer without a display (Actigraph wGT3x-BT) during all training sessions. For exercise training sessions, heart rate data is transferred to the accelerometer by Bluetooth and stored locally. To assess physical activity behaviour, patients are instructed to wear the accelerometer four days a week during daytime, of which at least one day during weekends. They upload exercise training and physical activity data to the web application weekly, by connecting the accelerometer to their personal computer using a USB-connection. On the web application, patients and their physical therapist (as well as other people who have access to the platform) can review the training and physical activity data graphically. A weekly video consultation with the physical therapist takes place via the web application.

These consultations are based on semi-structured interviews, in which the principles of motivational interviewing are applied. Motivational Interviewing is a psychosocial intervention technique to approach people engaged in behavioural change and tries to expose ambivalence between current and future behaviour [28–30]. The goal of Motivational Interviewing is to increase patients’ intrinsic motivation and encourage self-efficacy, in order to establish and maintain behavioural change.

During a video consultation, the physical therapist first checks whether the training sessions lead to symptoms, injuries or adverse events. Second, the physical therapist verifies whether patients adhere to their training schedule. Training frequency, session duration and intensity are recorded on the web application and are evaluated during the consultation. Together with the physical therapist, patients can adjust their training schedule if a structural problem prevents them from adhering to their schedule. Third, the physical therapist and patient review the physical activity data. They evaluate whether the physical activity targets have been reached (i.e. a desired amount of minutes spent at a moderate or high activity level). During the video consultation, the physical therapist identifies possible motivational issues, which can be exposed and addressed using motivational interviewing. We expect a video consult will take between 10 and 20 min.

#### Follow-up and on-demand coaching

During the final video consultation, the training period is evaluated and the individual exercise and physical activity targets are updated. These targets are recorded in the web application. Patients are instructed to use the heart rate monitor and accelerometer during the entire study period and to upload exercise training and physical activity data to the web application every week.

After the evaluation of the CR programme, coaching is performed on-demand. The web application evaluates the exercise training and physical activity data every four weeks and generates an alert in the following circumstances:

- Non-compliance: not uploading sensor data for ≥ 4 weeks.
- Reduced exercise training: a decrease of ≥ 50% in the time spent in a specified heart rate zone (average per week)
- Reduced physical activity: a decrease of ≥ 50% in the time spent in moderate to vigorous activity zone (average per day).

If an alert is generated, a video consultation is scheduled. In this video consultation, exercise and physical activity targets are evaluated and adjusted similarly to previous video consultations. If the targets are adjusted, the patient updates the targets in the web application.

### Outcome measures

**Fig. 1:**
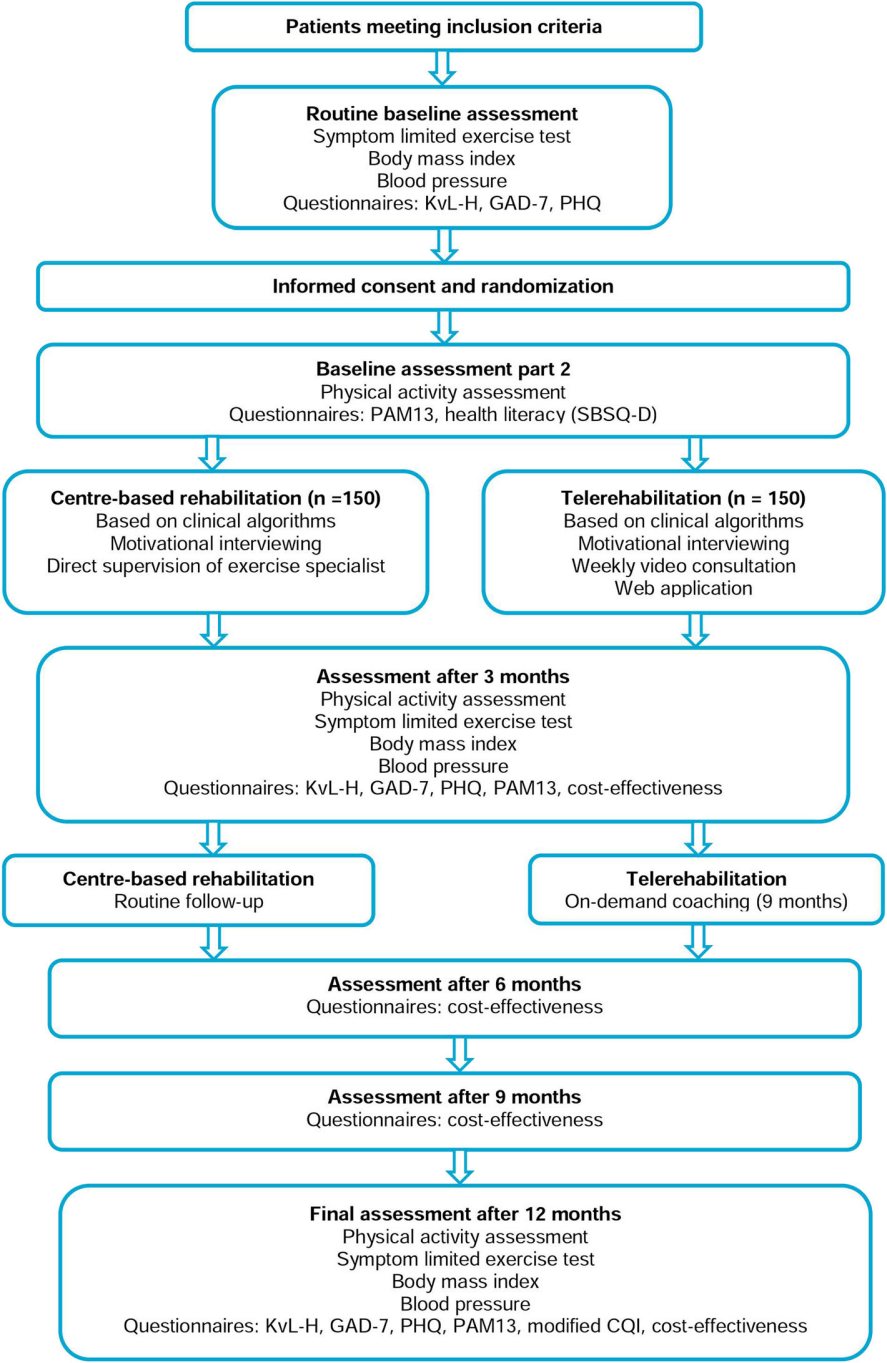
Flowchart of the study design. *KvL-H* Kwaliteit van Leven bij Hartpatiënten, *GAD-7* Generalized Anxiety Disorder, *PHQ* Patient Health Questionnaire, *PAM* Patient Activation Measure, *SBSQ-D* Set of Brief Screening Questions - Dutch, *CQI* Consumer Quality Index

The primary outcome measure is physical activity level, assessed at baseline, three months and twelve months. Secondary outcome measures are physical fitness, Body Mass Index (BMI), blood pressure, health related quality of life, anxiety and depression and patient empowerment, assessed at baseline and at three and twelve months. Other secondary outcome measures include patient satisfaction (assessed at twelve months) and cost-effectiveness (assessed at three, six, nine and twelve months). Health literacy is only assessed at baseline. An overview of the study design is provided in Fig. 1.

### Measurements

#### Physical activity level

Physical activity level (PAL) is determined by physical activity energy expenditure (PAEE), as described previously [31]. PAL is calculated from data from the accelerometer (ActiGraph wGT3x-BT) and a chest-strap heart rate monitor without a display (Polar H7, Polar Electro Oy, Kempele, Finland). The data of the heart rate monitor are transmitted to the accelerometer through Bluetooth. Patients are required to wear the sensors during daytime for an assessment period of four days, which includes two weekend days. During these days, the wrist-worn heart rate monitor (Mio Alpha) cannot be worn. Patients are blinded for both physical activity (accelerometry) and heart rate data.

After the assessment period of four days, patients in the intervention group upload the data to the web application. Patients in the control group return their sensors to the investigator. Accelerometry data (40Hz sample frequency) are synchronized with heart rate data (1Hz sample frequency) and resampled into one minute epochs. The energy expenditure estimation model developed by Kraal et al. is applied to calculate PAL [31].

#### Physical fitness

Physical fitness is assessed by a symptom limited exercise test. The maximal workload (in Watts) and percentage of the predicted value are calculated after completion of the test. The test will be performed on a cycle ergometer (Lode Corrival, Groningen), using an individualised ramp protocol aiming at a total test duration of 8–12 min. Patients are instructed to maintain a pedalling frequency of 70 rounds per minute. A twelve lead ECG is registered continuously.

#### Body mass index and blood pressure

The investigator measures length, weight and blood pressure prior to the symptom limited exercise test. Body mass index is defined as bodyweight divided by the square of ones height (in kilograms per square metre). Blood pressure is recorded using an automatic digital sphygmomanometer, using a standard cuff size for an arm circumference of 22–32 cm as well as an oversized cuff for larger arm circumferences according to the manufacturer's guidelines. Patients are instructed to adopt an upright sitting posture with the right arm on a table, the palm facing upwards, with uncrossed legs and the feet placed on the floor throughout the procedure. The cuff is applied to the right upper arm so that the bottom edge of the cuff is positioned 1–2 cm above the elbow joint and the cuff at heart height. At least two measurements are taken with a 5-min interval and the second blood pressure reading is used in the data analysis [32].

#### Health-related quality of life

Health-related quality of life is assessed by the KvL-H (Kwaliteit van Leven bij Hartpatiënten; *quality of life in cardiac* *patients*) questionnaire, a validated Dutch translation of the MacNew heart disease health-related quality of life questionnaire [33]. The questionnaire provides an overall score as well as scores on physical, emotional and social domain scales.

#### Anxiety and depression

The GAD-7 (Generalized Anxiety Disorder) and PHQ-9 (Patient Health Questionnaire) are recommended by the Dutch Society of Cardiology to screen CR patients for anxiety and depression [23]. The GAD-7 is a validated seven-item anxiety scale for the screening and assessment of generalized anxiety disorder [34]. The PHQ-9 is a 10-item multipurpose questionnaire for the screening and assessment of depression [35].

#### Patient empowerment, patient satisfaction and health literacy

Patient empowerment is assessed by the PAM13 (Patient Activation Measure) questionnaire [36]. This 13-item questionnaire was previously validated in the Dutch language and assesses patients’ skills for self-management of their health or chronic condition [37].

Patient satisfaction will be assessed by a modified version of the Dutch Consumer Quality Index (CQI) for rehabilitation medicine [38]. Health literacy is assessed by the Dutch version of the Set of Brief Screening Questions (SBSQ-D). This questionnaire consists of 3 questions with a 5-point Likert scale [39].

#### Cost-effectiveness

Both a cost-effectiveness analysis using PAL as effect measure and a cost-utility analysis using quality adjusted life years (QALYs) as outcome measure will be performed. The economic evaluation assumes a societal perspective, including both healthcare and non-healthcare related direct and indirect costs [40, 41]. Healthcare related costs include costs that are related to prevention, diagnostics and treatment; e.g. visits to healthcare professionals (outpatient visits, paramedical visits, general practitioner visits), assessment (exercise testing), professional wages, monitoring devices (accelerometer, heart rate monitor), medication and hospitalizations. Non-healthcare related costs include both costs made by the patient associated with the treatment, e.g. travelling costs and costs related to productivity loss due to absenteeism or presenteeism (the costs for productivity loss due to health issues while at work) in paid and unpaid work. Discounting in cost-effectiveness will not be used due to the time frame of the economic evaluation of one year.

Resource use in both treatment groups is prospectively assessed during the clinical study. Prices published in the Dutch Manual for Costing in economic evaluations and market prices are used [42]. Other costs are measured using the modified iMCQ (Medical Consumption Questionnaire) and iPCQ (Productivity Cost Questionnaire) questionnaires [40, 41], which patients fill out at three, six, nine and twelve months. To assess the effects of the delivery of informal care and to include these in the economic evaluation, informal carers of patients fill out the minimum variant of the iVICQ (Valuation of Informal Care Questionnaire) at three, six, nine and twelve months [43]. Either the friction cost method or the human capital approach is applied to determine the costs of absenteeism or inefficiency from paid work [44].

In the cost-effectiveness analysis, PAL is used as effect measure. In the cost-utility analysis, QALYs are measured using the EQ-5D-5L questionnaire at three, six, nine and twelve months [45].

### Statistical analysis

All analyses will be performed on an intention-to-treat basis. Descriptive statistics will be used to report demographics and baseline characteristics. Between-group differences and within group differences in the outcome measures will be evaluated using multivariate analysis of variance (MANOVA). Pearson’s correlation coefficient (r) will be applied to quantify relations between changes in variables. For all statistical comparisons, the level of significance will be set at p < 0.05. Analyses will be carried out in the statistical software package SPSS (version 22).

### Trial status

The Institutional Review Board of the hospital has approved the study protocol and its amendments prior to the start of the study. The inclusion of patients has started in May 2016 and is expected to be completed in September 2017.

## Discussion

The SmartCare-CAD trial is one of the first studies evaluating the effects and costs of a cardiac telerehabilitation intervention that combines modern technology (internet, sensor technology and video consultation) with evidence-based behavioural change strategies, including relapse prevention by on-demand coaching. The objective of this study is to investigate whether cardiac telerehabilitation using modern technology and multiple behavioural change strategies results in better long-term physical activity levels than centre-based CR in patients with CAD. We hypothesise that this intervention will result in improved self-management skills and a sustainable change in behaviour. This will translate to a superior increase in physical activity levels when compared to centre-based CR.

Based on the literature, it is still unclear how cardiac telerehabilitation compares to centre-based CR. Internet-based interventions may improve the efficacy of CR and can reach the majority of patients, with 81% of households in the European Union Member States – and 96% in the Netherlands – having internet access at home [46]. However, evidence to support internet-based delivery of CR is scarce [47, 48]. From the small amount of randomised controlled trials evaluating internet-based delivery of CR, only a few compare their intervention with centre-based CR [49–51].

Our study distinguishes itself from other studies by the use of modern communication technologies (internet, video consultation), relapse prevention by on-demand coaching, and coaching on both objective training intensity and physical activity. The assessment of both training intensity and physical activity in the home environment allows for individually tailored coaching for every patient for both components of the exercise program, instead of focusing on just one. Using the internet and video consultation may further enhance the effectiveness of the intervention [20]. Another important aspect of our study is the objective assessment of physical activity. As physical inactivity increases overall morbidity and mortality [52], an active lifestyle is considered as an important goal in CR. It is frequently reported in clinical studies, but often not objectively measured. Objectively measuring physical activity (or PAL) using accelerometer and heart rate data is more reliable than using questionnaires for self-reported physical activity [53], which enables an accurate assessment of the primary outcome measure.

Although a cost-effectiveness analysis is essential for the implementation of a novel intervention, only few cardiac telerehabilitation studies have performed such an analysis. A study by Frederix et al. showed that a prolonged, internet-based comprehensive telerehabilitation programme in addition to conventional CR was cost-effective [54]. Kidholm et al. however found that a cardiac telerehabilitation programme as an alternative to centre-based CR was not cost-effective [55]. In the SmartCare-CAD study, we will perform a comprehensive cost-effectiveness analysis, also taking into account productivity losses in paid and unpaid work due to health issues.

### Limitations

This study has a number of limitations. First, both the control and intervention group may be heterogeneous with respect to training frequency and intensity, since the number of training sessions will vary among patients, based on the aforementioned clinical algorithms [26]. Especially within the intervention group, variation may arise when patients do not transfer exercise training to the home environment during the first 3 months due to safety issues or patient preferences. However, these patients have access to the web application and will receive on-demand coaching from three months onwards. It is unknown to which extent this variation within both groups affects our outcome measures. Yet, we have chosen this study design since it best reflects clinical practice when such an intervention would be implemented. Besides, this variation is unavoidable because a patient’s CR programme should be determined individually based on the individual patient and disease characteristics [26]. Second, due to the Hawthorne effect, we expect patients to be more physically active on the days they wear sensors for the physical activity assessment. We expect that the effect does not impair the comparison of physical activity levels between groups to a great extent, because the Hawthorne effect is expected in both groups.

## Conclusion

The SmartCare-CAD study will provide new insights in the effects of internet-based cardiac telerehabilitation. It will address the added value of modern methods of communication, such as a patient-centred web application and videoconferencing. Besides, it will evaluate the effects of relapse prevention by on-demand coaching, both for coaching of exercise training and physical activity. Finally, it enables a direct and comprehensive cost-effectiveness analysis of cardiac telerehabilitation compared to centre-based CR.

## Additional file

Additional file 1: SPIRIT 2013 Checklist: Recommended items to address in a clinical trial protocol and related documents*. (DOC 120 kb)

## Abbreviations

CABG: coronary artery bypass grafting
CAD: coronary artery disease
CQI: Consumer quality index
CR: cardiac rehabilitation
CT: continuous training
GAD: Generalized anxiety disorder
HIT: high-intensity interval training
HRR: heart rate reserve
ICT: Information and communication technology
KvL-H: Kwaliteit van Leven bij Hartpatiënten
LVEF: left ventricular ejection fraction
MANOVA: multivariate analysis of variance
MCQ: Medical consumption questionnaire
NTR: Netherlands trial registry
NYHA: New York heart association
PAEE: physical activity energy expenditure
PAL: physical activity level
PAM: Patient activation measure
PCI: percutaneous coronary intervention
PCQ: Productivity cost questionnaire
PHQ: Patient health questionnaire
QALY: quality adjusted life year
SBSQ-D: Set of brief screening questions – dutch
SMS: Short message service
SPSS: Statistical package for the social sciences
VICQ: Valuation of informal care questionnaire
